# Sex Hormones and Blood Pressure

**Published:** 1941

**Authors:** A. Guirdham

**Affiliations:** Medical Superintendent, Bailbrook House, Bath.


### SEX HORMONES AND BLOOD PRESSURE.

BY

A. Guirdham, D.M., B.Sc., D.P.M.,

*Medical Superintendent, Bailbrook House, Bath.*

---

The ubiquitous effects of the sex hormones œstradiol and testosterone are becoming increasingly known. Their relation to blood pressure has received inadequate comment. Levy Simpson and Schæfer both indicate that œstrogens will decrease menopausal hypertension. It is claimed that testoviron can either raise or lower the blood pressure by acting as a stabiliser in sympathetic-parasympathetic imbalance, but that it cannot alter the blood pressure in such conditions as hyperpiesis due to organic disease such as renal disease or arteriosclerosis. The brief notes given below may add a little to our not very extensive knowledge of an important subject.

The aim of this short essay is to demonstrate the effect of sex hormones in lowering the blood pressure in other than menopausal conditions and, more particularly, to show the antagonizing effect on blood pressure of testosterone and œstradiol in the same person.

**Case 1.**—A female patient, aged 78, was admitted in a state of restlessness and acute depression, suffering from hyperpiesis. Her blood pressure was 220/120, with gross arterial degeneration and enlargement of the left ventricle. She was put on Progynon (œstradiol) ointment, 2 inches daily (=0·14 mgm. of œstradiol). In a fortnight her blood pressure was down to 150/70. This reduction was accompanied by a very marked improvement in her mental condition. The Progynon was stopped. The patient's blood pressure remained in the neighbourhood of the above reading.

**Case 2.**—The patient was a woman of 53, suffering from melancholia. She was quiet, rational and depressed, with marked retardation. She was in a very poor physical state, and arteriosclerotic, the myocardium being particularly deteriorated. Her blood pressure was 164/84. Her peripheral circulation was very poor.

Four days after admission she was put on Progynon ointment, 4 inches (=0·28 mgm. of œstradiol) being rubbed into the skin of the abdomen daily. A fortnight later she was much better mentally and the dose was increased to 8 inches (=0·56 mgm. of œstradiol) daily. After nineteen days her mental improvement was still more marked. Her blood pressure had fallen to 105/65. The dose of Progynon was reduced to 8 inches every other day. Reduction of the dose of œstradiol had a rapid effect in raising the blood pressure, so that three days after the reduction of the dose the reading was 130/80. On the day on which this reading was taken the patient had received no ointment.

It was reported to me that the patient seemed much better on days when she received the ointment. This reduced dose of 8 inches on alternate days was continued a further six days, at the end of which time her blood pressure, taken on a day when the ointment was administered, was 108/60. It seems possible that in addition to the undoubted mental improvement it caused over a long period, the ointment was a particularly rapid mental stimulant on the days on which it was administered—when a régime of alternate days was adopted—and that associated with the immediate stimulant effect was a fall in blood pressure.

Thirty-one days after commencing treatment with Progynon ointment her blood pressure had fallen from 164/84 to 96/60. The patient remained very well mentally and was not distressed physically. For the next month she had no hormone therapy and during that time her blood pressure remained low, though with the cessation of treatment it had risen to 108/60 at the end of the month. She was then put on Perandren (testosterone) ointment. She received 2 inches ( = 2 mgm. testosterone Ciba) daily. At the end of a week she was much better mentally, being brighter and infinitely more dynamic, and her blood pressure had risen to 130/70. The dose of Perandren was then doubled ( =4 mgm. testosterone) and after four days at the increased dosage the reading was 148/70.

These two cases are not isolated examples. They are chosen as representative of two kinds of reaction. It is regrettable that time and circumstances necessitate extreme condensation of these records. The remarkable action of œstradiol needs a great deal more investigation. It is too early yet for extravagant claims, but the existence of an agent capable of producing a gross reduction in a high blood pressure of organic causation is obviously of the first importance in medicine.

In addition to the above cases, I have noted marked reduction in blood pressure after treatment with œstradiol by inunction in female psychoneurotic patients of 30 and 40. This reduction was of a degree quite inexplicable on the grounds of diminution in anxiety, etc. Abnormalities of blood pressure among psychoneurotic patients are notoriously atypical, though I think not so markedly variable as has been claimed. In the cases I have in mind, the cause of the abnormal blood pressure could not be explained on these grounds.

It must be pointed out very definitely that I am not offering the thesis that œstradiol invariably lowers and testosterone inevitably raises the blood pressure. I claim no more than to have unmistakeably demonstrated this effect in certain patients. As a result of the many female cases I have treated with œstradiol either by mouth, injection or inunction, I believe that where œstradiol has any effect on blood pressure (and there are many cases in which none is noticeable), it acts in reducing the pressure. But the direct converse is far from true in cases treated with testosterone. In both male and female preclimacteric cases testosterone has caused a marked fall in blood pressure. Were the blood pressure in these cases excessive one might have thought that the testosterone was acting as a stabiliser of the vegetative nervous system, but the further reduction observed occurred in individuals with subnormal blood pressures.

### Summary.

Œstradiol can reduce a high blood pressure not only in menopausal hyperpiesis, but in conditions of raised pressure associated with arteriosclerosis and renal disease. It has also an effect in markedly lowering blood pressure in cases where the original pressure is not raised. It is possible in the same individual to lower and to raise the blood pressure at will by the administration of œstradiol and testosterone respectively. While all the evidence goes to show that where œstradiol affects the blood pressure at all, it does so by lowering the pressure, there is no such simple generalized tendency to be ascribed to testosterone.

